# Evaluation of the Psychosocial Treatment of Psychosis

**DOI:** 10.1192/j.eurpsy.2025.2148

**Published:** 2025-08-26

**Authors:** L. Mehl-Madrona, B. Mainguy

**Affiliations:** 1 Native Studies, University of Maine, Orono; 2 Psychiatry Residency, Northern Light Acadia; 3 Wabanaki Health and Wellness, Bangor, United States

## Abstract

**Introduction:**

Some people with a diagnosis of psychosis wish to minimize or avoid medications. A literture exists that intensive psychosocial treatment can mitigate psychotic symptoms with lilttle or no medication being used.

**Objectives:**

We provided services to people who wishers to reduce or avoid medications within the context of a private psychiatric practice and wanted to assess their outcomes. We wondered what factors led to success.

**Methods:**

We report on a series of 62 patients, age 18 years or older, who engaged in psychotherapy, medication, and lifestyle management over at least six months, aiming to minimize or eliminate medication. An additional 217 patients who consulted us did not continue for six months. An anonymous, matched comparison group of 62 patients of the same age, socioeconomic status, diagnosis, and severity of illness was generated from electronic health records at another clinic where LMM also worked. We used the Brief Psychiatric Rating Scale, the Positive and Negative Symptom Scale, the MADRS depression scales, and the Clinical Global Inventory. Narrative interviews generated qualitative data. We compared patients who met their goals to those who did not.

**Results:**

Forty-one people eliminated medication. Another 16 managed well on low-dose medications. Five patients had psychotic episodes that led them to return to higher levels of medication. This group functioned at higher levels than the comparison population with much lower doses of medications. The five readmissions to hospital were signnificantly lower than the number of readmissions in the comparison and the control groups. The cost for one year of care was higher for our people; the costs over subsequent years were less related to fewer hospitalizations, crises, and diminished suicidality.

**Conclusions:**

The results suggest the need for individualized client-centered psychosocial approaches that build upon the person’s previous successes, enroll family and friends in a community effort, and collaborate with those communities to apply those approaches desired by the people themselves. In this dialogical approach to psychosis, lived experience is granted full ontological reality, which appears to facilitate recovery. Lifestyle management and embeddedness in a community facilitate recovery.

**Disclosure of Interest:**

None Declared

